# Neutrophil-Derived MRP14 Supports Plasma Cell Commitment and Protects Myeloma Cells from Apoptosis

**DOI:** 10.1155/2019/9561350

**Published:** 2019-02-18

**Authors:** Zhihong Zhao, Zhonghua Luo, Qian Yang, Hongbo Chang, Pengcheng Liu, Zhichao Li, Shengli Guo, Chunhui Zhou, Jian Song, Weidong Cao

**Affiliations:** ^1^Department of Orthopedics, Beijing Second Hospital, Beijing 100031, China; ^2^Department of Interventional Radiology, Tangdu Hospital, Fourth Military Medical University, Xian 710038, China; ^3^Department of Natural Medicine, School of Pharmacy, Fourth Military Medical University, Xian 710032, China; ^4^Department of Neurosurgery, Navy General Hospital, Beijing 100048, China; ^5^Department of Neurosurgery, Yulin No. 2 Hospital, Yulin 719000, China; ^6^Institute of Physiological Chemistry and Pathobiochemistry, University of Muenster, Muenster 48149, Germany

## Abstract

Neutrophils have recently been proposed as cells with high functional plasticity and are involved in the pathogenesis of infections, malignancy, and autoimmune diseases. However, less is known about the role of neutrophil in humoral response. In this study, we examined the importance of neutrophils and the neutrophil-derived DAMP protein, MRP14, in antibody production. Splenic neutrophils and MRP14 that are present in the splenic peri-MZ region have a close contact with MZ B cells and promote their differentiation into plasma cells. Using neutrophil-depleting mice and an MRP14-blocking compound, we showed that the presence of neutrophil and MRP14 is required for class switch, plasma cell maintenance, and antibody production in the spleen. We found that MRP14 could also be produced by neutrophils in the bone marrow and support the maintenance of bone marrow plasma cells. MRP14 binding could enhance the effect of the BAFF signal and protect primary multiple myeloma cells from doxorubicin-induced apoptosis. Our data demonstrate the effects of neutrophils on neighboring B cells and plasma cells, which provides new insights into the connection between neutrophil and humoral responses.

## 1. Introduction

Neutrophils are known as the first wave of immune response to infection and inflammation. At the time of infection, neutrophils can be mobilized in large quantities from the bone marrow. In addition to bone marrow, neutrophils are also abundant in the lung and in the spleen. Recent studies have also shown that neutrophils residing in different tissues have different developmental stages or subtypes. In a tumor environment, for example, neutrophils can be polarized into the anti-tumor N1 cells and the pro-tumor N2 cells [1]. Neutrophils in the spleen can also be divided into immature and mature cells, both of which play an important role in clearing the blood-borne pneumococci [2].

In addition to the elimination of microorganisms and necrotic cells, new functions of neutrophils have been recently discovered in the regulation of humoral response. Spleen neutrophils can act as B helper cells, providing signals to spleen marginal zone (MZ) B cells, thereby inducing antibody production [3]. MZ B cells are the subpopulations of B cells located at the border of the spleen white pulp and red pulp, which are particularly important for rapid humoral immune defense against blood-borne pathogens [4]. Previous studies reported that MZ B cells are sensitive to the environmental milieu and their compartment and function are largely dependent on the interaction with the niches and the neighboring cells [5]. For example, splenic neutrophils can crosstalk with MZ B cells by producing cytokines such as BAFF, APRIL, and IL-21, triggering B cell class switch recombination and inducing T cell-independent antibody responses [6]. On the other side, the maintenance of MZ B cell function is also highly dependent on the signal transmitted by the Toll-like receptor (TLR), including pathogen-associated molecular pattern (PAMP) or damage-associated molecular pattern (DAMP) signals obtained in the microenvironment [7]. Different from follicular B cells, MZ B cells are characteristic not only of the polyreactive BCRs that bind to multiple molecular patterns and but also of the pronounced high expression of TLRs, allowing them to connect the innate and adaptive immune systems [8].

Neutrophils that are located in the spleen and bone marrow are in close contact with MZ B cells and plasma cells. Neutrophils can sense PAMP- and DAMP-TLR signals and further transduce these signals to related macrophages [9] and possibly to B cells and plasma cells. Being the terminally differentiated B cells, plasma cells also have a characteristic surface expression of TLRs, and the engagement of TLRs in plasma cells enhances their antibody production [10]. TLR ligation enhances the transcriptional level of Blimp-1 and XBP-1 and helps in the differentiation of MZ B cells into mature plasma cells [11]. In the study of systemic lupus erythematosus (SLE), activation of TLR4 has been shown to promote autoreactive plasma cell responses and enhance autoantibody production [12]. Studies of SLE have also shown that TLR signaling may act synergistically with BAFF through the TLR-associated signaling adaptor MyD88, which determines the proinflammatory isotypes of the autoantibody [13]. On plasma cells, dysregulated TLR stimulation leads to the production of type I interferons and uncontrolled cell proliferation, which is independent of MyD88 and is often associated with the development of multiple myeloma [14].

Recently, MRP14 has been identified as the key DAMP molecule and the endogenous ligand of TLR-4 [15]. It has been reported that MRP14 is released by activated phagocytic cells and has a proinflammatory effect on vascular injury, phagocytosis, and development of autoreactive CD8 T cells [7–9]. However, the role of MRP14 in humoral responses remains unknown. In the current study, we demonstrate that neutrophils inhabiting the peri-MZ region of spleen specifically produce MRP14. Spleen neutrophils and their derived MRP14 are required for promoting MZ B cell proliferation, class switching, and spleen plasma cell maintenance. MRP14 could also be produced by neutrophils in the bone marrow, which supports the maintenance of plasma cells. MRP14 binding could enhance the effect of the BAFF signal and protect primary multiple myeloma cells from doxorubicin-induced apoptosis. Our data show that neutrophils transduce DAMP signal molecule MRP14 and trigger the TLR signaling pathway, which is required for the maintenance of MZ cell B cells and plasma cells.

## 2. Material and Methods

### 2.1. Mice

6 to 12-week-old mice (C57BL/6) were obtained from the Jackson Laboratory. Experiments were conducted according to Chinese Animal Welfare guidelines. During the experiments, the mice with skin wound or weight loss over 10% are excluded.

### 2.2. Antibodies

Antibodies for immunofluorescence studies were as follows: rat anti-mouse IgM (553437, Pharmingen), MOMA-1 (T2011, BMA Biomedicals), MARCO (MCA1849, Serotec), and rat anti-mouse MRP14 (565833, BD Biosciences). Secondary antibodies included goat anti-rabbit FITC-conjugated IgG, rhodamine-conjugated IgG (H + L) (111-225-045, 111-165-144, Dianova), Cy5-conjugated IgG (111-605-144, Dianova), goat anti-rat Alexa Fluor 568 IgG (A11077, Invitrogen), and donkey anti-rat Alexa Fluor 488 IgG, (A21208, Invitrogen).

Antibodies to the following were used in flow cytometry analyses to identify cellular compartments: PE- or APC-labeled anti-mouse Ly6G (127607, 127614, BioLegend), APC-labeled anti-mouse CD11b (17-0112-83, eBioscience), biotin- or PE-labeled anti-mouse CD23 (553139, Pharmingen), FITC- or PE-labeled anti-mouse CD21 (552957, 553818, Pharmingen), FITC-labeled anti-mouse BrdU (559619, BD Biosciences), PerCP-labeled anti-mouse CD19 (552854, Pharmingen), rat anti-mouse MRP14 (565833, BD Biosciences), FITC-labeled anti-mouse BAFF receptor (11-5943, eBioscience), and PE-labeled anti-human BAFF receptor (316906, BioLegend). Human neutrophils and plasma cells were detected using FITC-labeled anti-human CD66b (305103, BioLegend) and APC-labeled anti-human CD138 (352308, BioLegend).

### 2.3. Neutrophil Depletion

Neutrophils were depleted by a single intravenous injection of 100 *μ*g of anti-Ly6G (127602, BioLegend, 1A8) every two days. Blood cells were analyzed by flow cytometry to assess the efficiency of depletion. More than 90% of the circulating neutrophils were depleted 24 hours after anti-Ly6G injection. Control animals were injected with the isotype control antibody rat IgG2a, wherein the neutrophil population did not change compared to the nontreated mice.

### 2.4. Immunization/Antibody Response

Mice were injected intravenously with 50 *μ*g of NP-Ficoll dissolved in a volume of 200 *μ*l of PBS. One week after immunization, serum IgG3 and IgM titers were measured using the Clonotyping System Kit (5300-05, Southern Biotech).

### 2.5. MRP14 Blocking Assay

Paquinimod (ABR-215757) is an immunomodulatory compound preventing MRP14 binding to TLR-4 [16]. Starting at 3 days before the immunization, mice were intraperitoneally injected daily with 25 *μ*g paquinimod (319595, MedKoo) that dissolved in PBS (10 *μ*g/g body weight), which lasts till the end of the experiment.

### 2.6. Immunofluorescence Histology

Spleens were embedded in Tissue-Tek and then frozen at -70°C. Tissues were cut into 10 *μ*m thick sections using a cryostat. Bone marrows that were punched out with a syringe (No. 7 needle) with a Tissue-Tek were frozen and sectioned. Before staining, sections were fixed in methanol at -20°C for 5 minutes. Sections were incubated with primary antibodies overnight at 4°C and proceeded with the respective immunofluorescent labeled secondary antibody for 1 hour. Specimens were examined using a Zeiss Axio Imager microscope equipped with epifluorescent optics. Images were analyzed using ImageJ software.

### 2.7. Flow Cytometry

Mouse spleen cells were obtained after spleens were sieved through a 70 *μ*m filter. Red blood cells were lysed with red blood cell lysis solution (349202, BD Biosciences). B cell subsets were analyzed using FACSCalibur (BD Biosciences) and determined with antibody staining, including CD19^+^CD21^hi^CD23^lo^ MZ B cells, CD19^+^CD21^lo^CD23^hi^ follicular B cells, CD19^−^CD138^+^ plasma cells, and Ly6G^hi^CD11b^hi^ neutrophils. Expression of the BAFF receptor and the surface-bound MRP14 were determined using the antibodies listed above.

### 2.8. qPCR

CD19^+^CD21^hi^CD23^lo^ MZ B cells were isolated from adult spleens using FACSAria (>98% purity). qRT-PCR was carried out on sorted MZ B cells for gene expression of AID (*AICDA*) and Blimp1 (*PRDM1*). The primers used for qRT-PCR were *AICDA*, forward primer 5′-GCCACCTTCGCAACAAGTCT-3′, reverse primer 5′-CCGGGCACAGTCATAGCAC-3′, *PRDM1*, forward primer 5′-AAGAGGTTATTGGCGTGGTAAG-3′, reverse primer 5′-TAGACTTCACCGATGAGGGGT-3′.

### 2.9. *In Vivo* Proliferation Assay

Splenic B cell proliferation was quantified by BrdU incorporation. Briefly, 2 mg of BrdU in 200 mg PBS was injected intravenously 16 hours prior to sacrifice of the immunized mice. The incorporated BrdU is detected using a BrdU Flow kit (559619, BD Pharmingen) and analyzed by flow cytometry in combination with MZ B cell surface markers.

### 2.10. Isolation of Human Neutrophils

EDTA anticoagulated blood was obtained from healthy volunteers. Neutrophils were isolated using MACSxpress® whole blood neutrophil isolation kit (130-104-434, Miltenyi Biotech) according to the manufacturer's instructions. Afterwards, erythrocytes were lysed using the red blood cell lysis solution (349202, BD Biosciences). The purity of neutrophils was assessed by flow cytometry and was >95%.

### 2.11. Apoptosis Assay

Bone marrow cells were obtained from patients with multiple myeloma. Plasma cells were isolated using the human plasma cell isolation kit (130-093-628, Miltenyi Biotech) according to the manufacturer's instructions. The purity was assessed by flow cytometry and was >90%. Cells were cultured in RPMI 1640 medium containing 10% FCS and 2 mM L-glutamine at 37°C. To measure the surface expression of the BAFF receptor, neutrophils were cocultured with plasma cells or recombinant human MRP14 protein (5 mg/mL, 9254-S9-050, R&D). For the apoptosis assay, myeloma plasma cells were treated with doxorubicin (0.4 *μ*g/ml, Sigma) for 24 hours and assessed by flow cytometry with FITC-labeled Annexin V (556547, BD Biosciences). Recombinant human BAFF protein (100 ng/mL, 2149-BF-010, R&D) was used to rescue the plasma cells from apoptosis.

### 2.12. Statistical Analysis

Quantitative data are shown as means ± SEM. Significance of antibody titers, relative expression, and cell numbers was analyzed with unpaired Student *t* test 5that was performed using Prism (GraphPad). The *P* values <0.05 were considered significant.

## 3. Results

### 3.1. Splenic Neutrophils in the Peri-MZ Region

**Figure 1:**
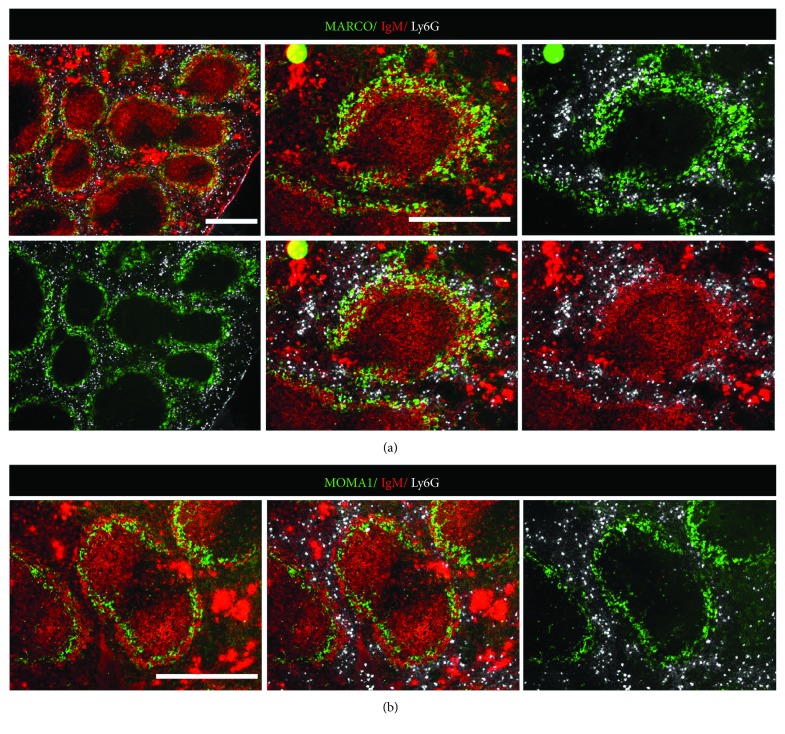
Neutrophils correlate with the MZ B cell region in the spleen. *Immunofluorescence staining of adult spleen sections*. (a) Anti-MARCO stains the MZ macrophages, correlated with the area of IgM high-expressing MZ B cells. The Ly6G antibody marks the splenic neutrophil in the peri-MZ region, surrounding the white pulp (WP) and proximal to the MZ B cells and macrophages. Pictures are shown in overview of WP and high magnification of the MZ structures. (b) Anti-MOMA-1 labels the metallophilic macrophages, which locates at the inner ring of the MZ region and rarely in touch with spleen neutrophils. Scale bars represent 100 *μ*m. Data represent two independent experiments with two mice each experiment.

One of the main roles of the spleen is to make antibodies that protect the body against blood-borne viruses, bacteria, and other microorganisms [17], which are secreted by B cells after exposure to antigens. In contrast to follicular B cells that are situated within the follicles, MZ B cells are continuously exposed to the blood, which react quickly to incoming pathogens and therefore represent an important first line of immune defense. To delineate the interaction of spleen MZ B cells and their neighboring cells, we performed tissue immunofluorescent analyses in the peri-MZ region. Using the MARCO antibody which permitted the spatial analyses of MZ macrophages, we can see the overlapping distribution of MZ B cells and MZ macrophages (Figure 1(a)). Using neutrophil antibody Ly6G, we could detect spleen neutrophils in the blood-filled red pulp, which have a close contact with MZ B cells and macrophages. This proximity in position indicates a connection of MZ B cells to the blood-circulating neutrophil in the spleen. In contrast, spleen neutrophils maintain a distance from the follicular B cells in the follicle. Staining of MOMA-1+ metallophilic macrophages marks the border between MZ and follicular B cells (Figure 1(b)).

### 3.2. Spleen Neutrophils Involved in Plasma Cell Differentiation and Maintenance

**Figure 2:**
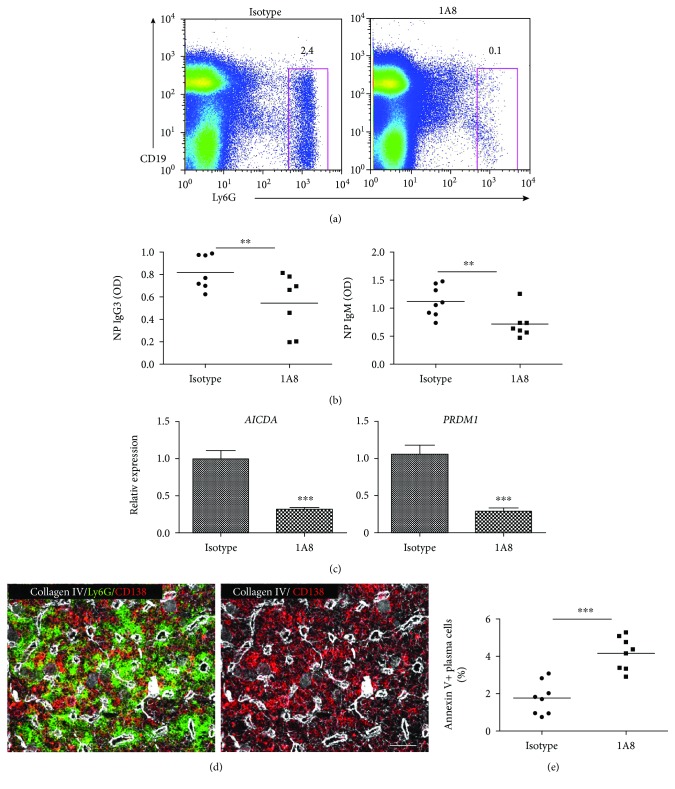
Splenic neutrophils help in the immune response of MZ B cells. (a) Neutrophils were depleted by injecting 1A8 monoclonal antibody, and the depletion efficiency was measured using flow cytometry analysis. After intravenous immunization with T cell–independent antigen NP-Ficoll, (b) serum levels of NP-specific IgG3 and IgM in the neutrophil-deficient mice were determined using ELISA. (c) qRT-PCRs reveal the mRNA levels of AID (*AICDA*) and BLIMP-1 (*PRDM1*) for the isolated MZ B cells from the immunized neutrophil-sufficient or -deficient mice. (d) Ly6G-positive neutrophils are shown to associate with the plasma cells (CD138+) in the bone marrow. (e) After neutrophil depletion, the apoptosis in the bone marrow plasma cells were assessed using Annexin V staining. Data shown are means ± SEM from at least seven mice for each group and pooled from three independent experiments. ^∗∗^*P* < 0.01 and ^∗∗∗^*P* < 0.001.

To further study the impact of spleen neutrophils on the MZ B cell immune responses, we depleted neutrophils by systemic injection of 1A8 mAb (Figure 2(a)) and examined the spleen B cell compartment and the TI antigen response. Neutrophil-deficient mice demonstrated an intact MZ B cell compartment (not shown), but showing a reduced level of antibody production of NP-specific of IgG3 and IgM (Figure 2(b)). This reduction was associated with the downregulation of the class switch recombination- (CSR-) inducing enzyme activation-induced cytidine deaminase (AID) and the plasma cell-inducing transcription factor *PRDM1* (the gene encoding the plasma cell–associated proteins, Blimp-1) (Figure 2(c)), which are measured using qPCR analysis of the isolated MZ B cells from the neutrophil-depleting mice. Taken together, this data suggested that spleen neutrophils are required for MZ B cells to undergo CSR and plasma cell differentiation.

Besides the circulating neutrophils, a large pool of neutrophils is in the bone marrow, from which spleen neutrophils are derived, and to which plasma cells are also homing. The bone marrow is a niche important for both neutrophil homeostasis and antibody production. Using immunofluorescent staining, we performed spatial analysis in the bone marrow and observed a close contact between neutrophils and the CD138+ plasma cells (Figure 2(d)). To elucidate whether depletion of neutrophil has an impact on the plasma cells in the bone marrow, we assessed the bone marrow plasma cells by flow cytometry analysis of apoptotic cells using Annexin V staining. An increased apoptotic rate was found in the plasma cells from neutrophil-deficient mice compared to neutrophil-sufficient mice (Figure 2(e)), but no change was detected in the populations of circulating B cells and B cell progenitors (not shown). This data suggested that bone marrow neutrophils might play a role in plasma cell maintenance.

### 3.3. Splenic and Bone Marrow Neutrophil Secreting DAMP Cytokine MRP14

**Figure 3:**
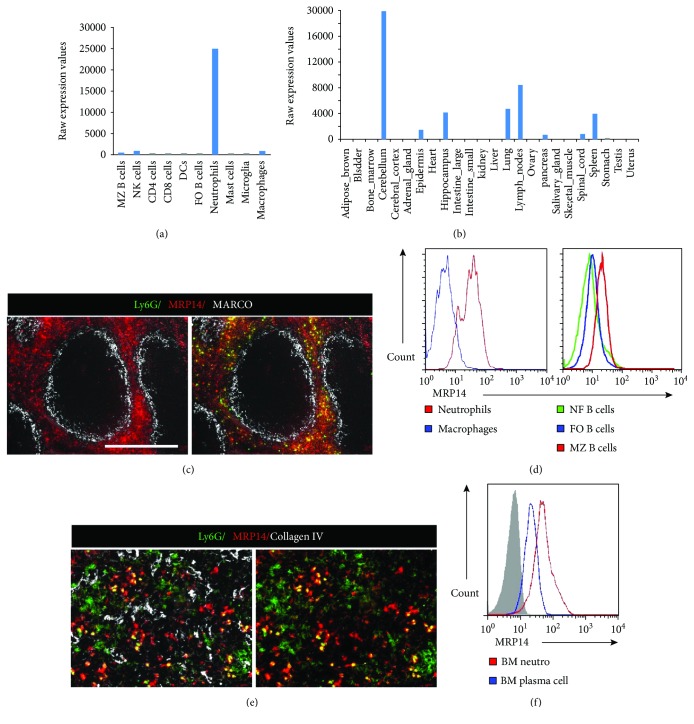
MRP14 expression in the splenic neutrophils and correlation with MZ B cell. Expression of MRP14 mRNA in (a) immune cells and (b) various mouse tissues. The data are from BioGPS and are presented as raw expression values. (c) Anti-MARCO stains the MZ macrophages, and MRP14 staining exhibits in the spleen peri-MZ region. Costaining with Ly6G antibody shows the highly concentrated MRP14 on the splenic neutrophils. Scale bars represent 100 *μ*m. (d) Flow cytometry histograms show that substantial amounts of MRP14 protein are present on the surface of splenic neutrophils and MZ B cells. (e) Anti-collagen type IV stains vessels and sinus in the bone marrow. Costaining shows the correlation between MRP14 staining and Ly6G+ neutrophils. Scale bars represent 100 *μ*m. (f) Flow cytometry histograms show the surface presence of MRP14 protein on the neutrophils and the plasma cells in the bone marrow. Data shown represent means at least six mice from three independent experiments.

Next, we questioned the molecules that could be produced by neutrophils and affect the immune response of MZ B cells and the maintenance of plasma cells. A survey of public databases (BioGPS and GEO Profiles) revealed that DAMP molecule MRP14 is specifically expressed by neutrophils (Figure 3(a)) and highly present in the neutrophil-abundant organ, including bone marrow, lung, and spleen (Figure 3(b)). Nevertheless, lymph nodes could also be noticed for the high expression of MRP14, which might be due to the nonneutrophil cells. To elucidate the expression pattern of MRP14, we performed tissue immunofluorescence analysis in the spleen and detected that MRP14 colocalizes and surrounds the Ly6G+ neutrophil cells, covering a large region of the peri-MZ zone that is adjacent to the MARCO+ MZ macrophages (Figure 3(c)). As MRP14 is expressed as both membrane-bound and of extracellular form and can be detected on the cell surface, we identified the substantial amount of surface-bound MRP14 using flow cytometry analysis. Neutrophils exhibited a higher level of surface MRP14 when compared to macrophages. MZ B cells also exhibited a higher level of surface MRP14 when compared to follicular B cells (Figure 3(d)). In the bone marrow, we found many MRP14-secreting neutrophils (Figure 3(e)), which is correlated with the substantial amount of surface-bound MRP14 on the bone marrow neutrophils and plasma cells (Figure 3(f)). Taken together, this data indicates that MRP14 is mainly produced and secreted by neutrophils, which might be present as a cell surface-bound molecule on their neighboring immune cells, such as MZ B cells in the spleen and plasma cells in the bone marrow.

### 3.4. MRP14 Promotes MZ B Cell Differentiation into Plasma Cells

**Figure 4:**
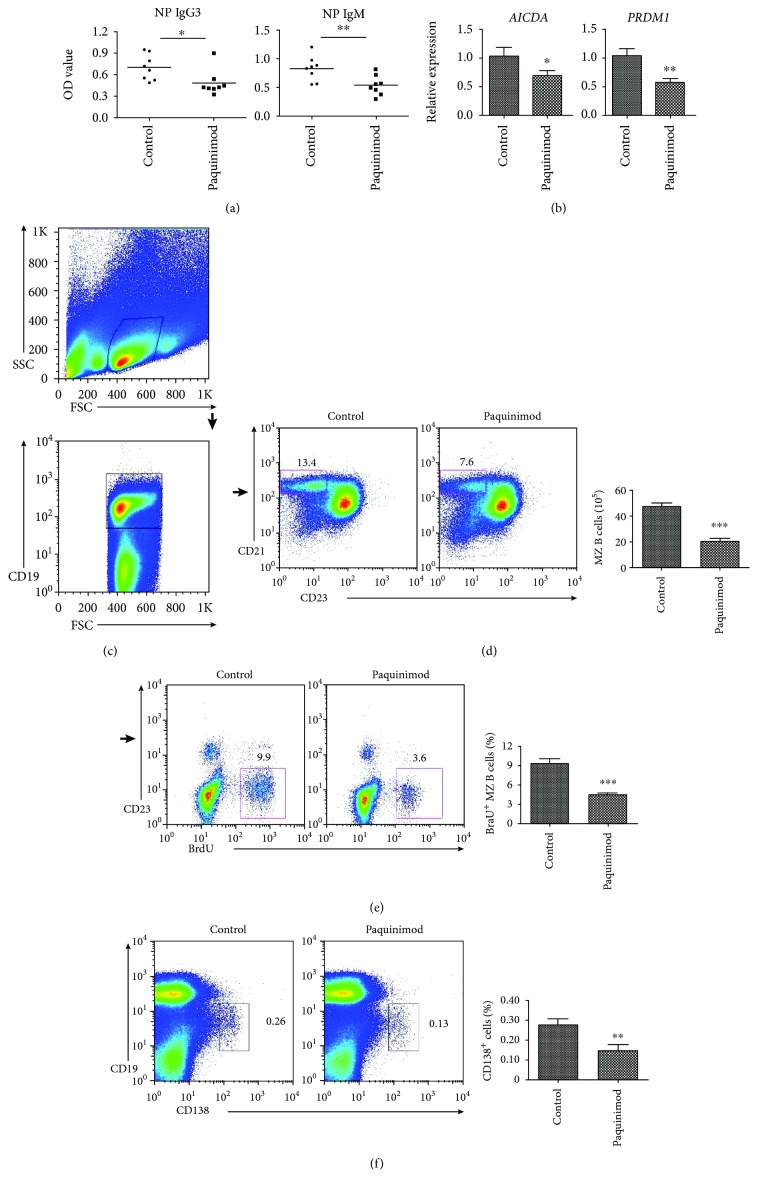
Blockade of MRP14 signal reduces the immune response of MZ B cells. (a) ELISA data reveals the serum levels of IgG3 and IgM in the paquinimod or PBS-injected mice in response to intravenous immunization with NP-Ficoll. (b) qRT-PCRs reveal the MZ B cell mRNA levels of AID (*AICDA*) and BLIMP-1 (*PRDM1*) from the paquinimod or control-treated mice one week after immunization. (c) Gated on splenic live CD19+ B lymphocytes, (d) frequencies and total numbers of MZ B were analyzed. (e) Flow cytometric quantification of BrdU+ frequency reveals the proliferation rate of CD19+CD23- MZ B cells in the spleens. (f) After paquinimod treatment, the population of CD138+ plasma cells in the bone marrow was determined. ^∗^*P* < 0.05, ^∗∗^*P* < 0.01, and ^∗∗∗^*P* < 0.001. Flow cytometric data shown represent at least six mice from three independent experiments.

To study the role of MRP14 in MZ B cell response, we injected mice with MRP14-blocking compound paquinimod [16] before and during the immunization of TI antigen. Similar to the neutrophil depletion experiment, paquinimod injection attenuated the NP-specific responses of IgG3 and IgM (Figure 4(a)) and downregulated the RNA level of AID and Blimp1 in MZ B cells (Figure 4(b)). The spleen from mice treated with paquinimod showed fewer MZ B cells compared to the sham-injected mice (Figures 4(c) and 4(d)). Also, blockade of MRP14 reduced BrdU incorporation in MZ B cells after NP-Ficoll immunization, as a result of the reduced cell proliferation (Figures 4(c) and 4(e)). In the bone marrow, paquinimod-treated mice exhibited a reduced population of CD138+ plasma cells compared to the controls (Figure 4(f)). This data suggests that MRP14 might also have direct impacts on the maintenance of the MZ B cell and plasma cell compartments.

### 3.5. MRP14 Protect Multiple Myeloma Cells from Apoptosis

**Figure 5:**
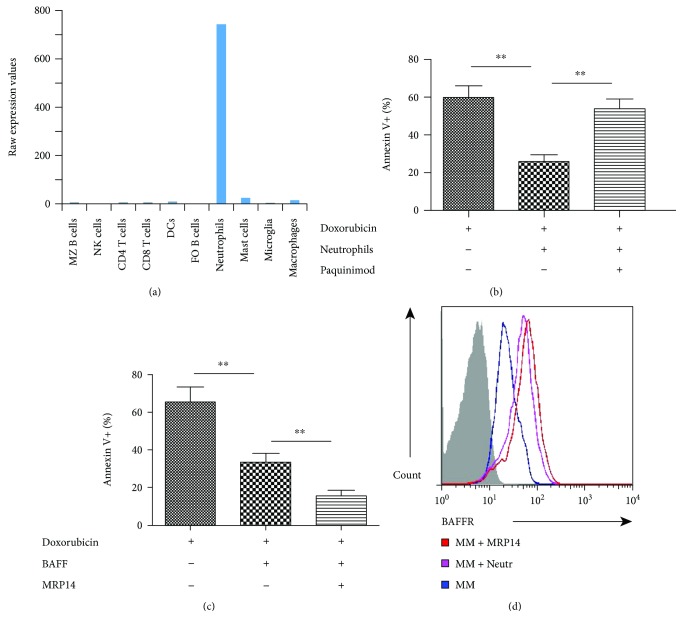
MRP14 protects multiple myeloma cells from apoptosis. (a) Expression of BAFF mRNA in immune cells. Data from BioGPS are presented as raw expression values. Myeloma plasma cells were cultured with doxorubicin for 24 hours (b) with neutrophils or (c) with BAFF plus MRP14. Annexin V-positive cells were shown for the apoptotic plasma cells. (d) Coculture with neutrophils or MRP14 enhanced the surface level of the BAFF receptor of myeloma cells, which is determined using flow cytometry. Data shown are means ± SEM and pooled from four independent experiments. ^∗∗^*P* < 0.01.

Surveying the cytokine production profile of neutrophils, we noticed that neutrophils produce a much higher level of BAFF, when compared to other immune cells (Figure 5(a)). BAFF is a crucial factor contributing to the maintenance of plasma cells and has been shown to be involved in the pathogenesis of multiple myeloma [18]. As neutrophils secrete both MRP14 and BAFF, we question whether the presence of MRP14 could impact on BAFF and lead to a synergized signaling that supports survival of plasma cells. We tested this hypothesis using primary multiple myeloma, as these cells are often associated with dysfunctional BAFF and TLR signaling. The isolated primary multiple myeloma cells were treated with doxorubicin, which could rapidly induce the apoptosis of myeloma cells. The in vitro cell culture experiments showed that while BAFF could efficiently protect the multiple myeloma cells from apoptosis, the presence of MRP14 could further decrease the proportion of Annexin V-positive cells (Figure 5(b)). When we cocultured the multiple myeloma cells with neutrophils, the number of apoptotic cells was also dramatically reduced, the effect of which could be reversed by adding the MRP inhibitor (Figure 5(c)). This data indicates that MRP14 might have a crosstalk with BAFF signal transduction and promote the BAFF-induced survival signal. Correlatively, we could show that the multiple myeloma cells upregulated the surface expression of the BAFF receptor, upon the stimulation by neutrophils or by MRP14 protein (Figure 5(d)).

## 4. Discussion

In the current study, we show that neutrophils that inhabit the peri-MZ region of the spleen and the perivascular region of bone marrow could produce an endogenous danger signal molecule, MRP14, which supports the maintenance of spleen MZ B cells and plasma cells.

Our experiment indicates that in addition to BCR stimulation, the MRP14 signal is required for the induction of AID and CSR in the B cells. It is known that the induction of AID and CSR involves both BCR and TLR, which reflects the activation of the noncanonical and canonical NF-*κ*B pathways [19]. BCR signaling activates the noncanonical NF-*κ*B pathway, and the TLR-dependent signal activates the canonical NF-*κ*B pathway. The activation of both pathways is necessary to provide a sufficient signal for the AID induction and class switch DNA recombination. During T-independent immune responses, LPS alone could rapidly induce MZ B cell migration and antibody production, as it possesses both polysaccharidic moiety and lipid A moiety, thereby triggering both TLR4 and BCR signaling. Unlike LPS, the MRP14 signal acts only through the TLR4 pathway and is constitutively expressed [20]. Therefore, we assume that MRP14 is synergized with the BCR signals that are triggered by basal antibodies and is critical for plasma cell maintenance and antibody production in the steady state.

Also, our data here show that MRP14 stimulation leads to the upregulation of receptors of BAFF, indicating a crosstalk between TLR and BAFF signaling. BAFF is known to be secreted by spleen B helper neutrophil and crucial for AID expression and extrafollicular CSR [21]. Belonging to the TNF family, BAFF signals through the noncanonical NF-*κ*B pathway, which contributes to B cell survival and differentiation and is also crucial for plasma cell maintenance and antibody production [22]. Although the BAFF receptor is mainly expressed on the surface of B cells, BAFF is secreted by many cell types that have a close contact with B cells, which indicates a tight connection between B cells and their neighborhood. We have shown here that neutrophils might impact on their neighboring B cells by producing not only BAFF but also MRP14. We also found that MRP14-induced TLR signaling might synergize the BAFF signal. It has been shown that BAFF-induced lupus-like phenotypes are largely dependent on the TLR-associated signaling adaptor, MyD88 [13]. Moreover, plasma cell commitment requires both the canonical NF-*κ*B signal and the noncanonical signal [19], which could be induced by TLR and BAFF, respectively. The combination of the MRP14-TLR signal and BAFF could also contribute to the maintenance of plasma cells, which correlated with the previous finding that TLR+ plasma cells show pronounced enhanced production of anti-dsDNA autoantibodies in the BAFF-dependent lupus [12].

Our data revealed a large amount of extracellular MRP14 in the spleen and bone marrow. In comparison with MZ and metallophilic macrophages, neutrophils dominated the expression of MRP14 in the MZ region of spleen. Although the transcriptional regulation of MRP14 by neutrophils remains elusive, it is known that extracellular MRP14 is regulated by MMP proteases, which might keep the homeostasis level of functional MRP14 [23]. Other than the spleen, MRP14 is detected in substantial level in another secondary lymphoid organ, lymph nodes [24]. Due to the small number of lymph node infiltrating neutrophils, we did not include here the investigation of lymph node-derived MRP14. However, the impact of MRP14 on the T cell-dependent antigen response in the lymph nodes is interesting and needs further investigation.

It has been shown that MRP14 binding triggers MyD88-mediated TLR4 signaling, leading to NF-*κ*B activation and secretion of proinflammatory cytokines [25]. Therefore, MRP14 has so far been suggested as a proinflammatory cytokine, being involved in the pathogenesis of systemic infections, malignancy, and autoimmune diseases. However, MRP14 is also involved in immune cell differentiation such as dendritic cells and erythroid cells and demonstrates the regulatory function on a certain disease phase [24, 26]. We show here that MRP14 could promote MZ B cell proliferation and the following CSR and antibody production. However, whether MRP14 is involved in the development of spleen MZ B cells is still under investigation.

Our data here showed that the neutrophil pool in the bone marrow produces a large amount of MRP14, which stimulates the TLR4-expressing plasma cells and has a significant impact on the maintenance of plasma cells. This observation is correlated with the studies in the multiple myeloma that the malignant plasma cells are associated with dysregulated TLR stimulation [27]. The high number of neutrophils often indicates a poor prognosis in the multiple myeloma patients. In the coculture experiment, we could show that the presence of neutrophil or MRP14 protects the myeloma cells from apoptosis. This protection might be attributed to BAFF signaling which is enhanced by MRP14-TLR signaling and is crucial for the survival of plasma cell myeloma [28].

Collectively, our findings indicate that only the spleen MZ B cells that got help of neighboring neutrophils can undergo the proper TLR-dependent maintenance and differentiation, in response to T-independent antigen. Furthermore, neutrophils crosstalk with plasma cells and protect plasma cells from programmed apoptosis. The malfunction of neutrophils or neutrophil-derived MRP14 could aggravate the pathogenesis of humoral response disorders or plasma cell myeloma. Our study provides a new insight for neutrophils in connecting innate immunity and humoral immune response.

## Data Availability

The immunostaining and flow cytometry data used to support our findings are included within the article. The gene expressing data and quantification data are available on request.
